# Non-Surgical Respiratory Management in Relation to Feeding and Growth in Patients with Robin Sequence; a Prospective Longitudinal Study

**DOI:** 10.1177/10556656231199840

**Published:** 2023-09-20

**Authors:** Pleun P.J.M. van der Plas, Gwen G.M. van Heesch, Maarten J. Koudstaal, Bas Pullens, Irene M.J. Mathijssen, Simone E. Bernard, Eppo B. Wolvius, Koen F.M. Joosten

**Affiliations:** 1 Department of Oral and Maxillofacial Surgery, Sophia Children's Hospital – 6993 Erasmus Medical Center, Rotterdam, The Netherlands; 2 Department of Pediatric Intensive Care, Sophia Children's Hospital – 6993 Erasmus Medical Center, Rotterdam, The Netherlands; 3 Department of Otorhinolaryngology, Sophia Children's Hospital – 6993 Erasmus Medical Center, Rotterdam, The Netherlands; 4 Department of Plastic and Reconstructive Hand Surgery, Sophia Children's Hospital – 6993 Erasmus Medical Center, Rotterdam, The Netherlands

**Keywords:** dysphagia, mandible, pediatrics, upper airway obstruction, craniofacial morphology, cleft palate

## Abstract

**Objective:**

To reflect upon our non-surgical respiratory management by evaluating clinical outcomes regarding airway, feeding, and growth during the first year of life in patients with Robin Sequence.

**Design:**

Prospective study.

**Setting:**

Sophia Children's Hospital, Rotterdam, the Netherlands.

**Patients/ Participants:**

36 patients with Robin Sequence who were treated between 2011 and 2021.

**Interventions:**

Positional therapy and respiratory support**.**

**Main Outcome Measure(s):**

Data on respiratory outcomes included polysomnography characteristics and capillary blood gas values. Feeding outcomes were based on the requirement of additional tube feeding. Outcomes on growth were expressed as standard-deviation-scores (SDS) for weight-for-age (WFA) and height-for-age (HFA).

**Results:**

Twenty patients were treated with positional therapy (PT), whilst the other 16 patients required respiratory support. Twenty-two patients presented with non-isolated Robin Sequence (RS). During the first year of life, obstructive apnea hypopnea index decreased, oxygen levels enhanced, and capillary blood gas values improved. Eighty-six percent (31/36) experienced feeding difficulties, which completely resolved in 71% (22/31) during their first year of life. From start treatment, to stop treatment, to the age of 1 year, the SDS WFA worsened from −0.40 to −0.33 to −1.03, respectively.

**Conclusions:**

Non-surgical respiratory treatment resulted in an improvement of respiratory outcomes to near normal during the first year of life in patients with RS. These patients often experience feeding difficulties and endure impaired weight gain up to 1 year of age, despite near normalization of breathing. The high prevalence of feeding difficulties and impaired weight for age indicate the urgency for early recognition and adequate treatment to support optimal growth.

## Introduction

Robin Sequence (RS) is a rare congenital craniofacial abnormality that occurs in around 1/5600 live births.^1^ It is characterized by a typical triad of symptoms, including mandibular hypoplasia (MH), glossoptosis, and tongue-based upper airway obstruction (UAO) (2). In 90% of the cases, a cleft palate (CP) is present.^2,3^ RS may occur as an isolated entity, but the majority of patients presents with other anomalies or a syndrome (non-isolated RS), with over 40 syndromes described being associated with RS.^4,5^ Besides the genotypic heterogeneity in RS, there is also a wide variability in clinical presentation.^6^ Closely related to UAO are feeding difficulties (FD), with or without concomitant swallowing difficulties, which are present in around 80% of the patients.^7^ Besides poor feeding skills due to anatomical abnormalities (MH, glossoptosis, CP) and/or (immature or permanent) disturbed swallowing mechanisms, gastro-oesophageal reflux, regurgitation, and vomiting are also highly prevalent in these patients, which further complicates adequate intake.^8^ In combination with the increased energy expenditure to work against an obstructed airway, additional tube feeding is often required to prevent malnutrition and delayed growth.^9–13^ Timely recognition and adequate treatment of the respiratory difficulties and feeding problems is essential to prevent serious comorbidities including failure to thrive, cardiovascular problems, increased risk of psychological problems, and in extreme cases sudden death, with mortality rates up to 20%.^14–18^

In literature, various treatment strategies to treat the UAO have been described, ranging from positional therapy to more invasive, surgical intervention.^19–21^ Although several management algorithms have been proposed, there is currently no widely accepted treatment protocol.^2,22^ Hence, the management of children with RS remains controversial and treatment protocols vary per center, depending on the institution's preferences.

Despite growing attention to RS, most studies only evaluated outcomes after surgical management for UAO, whilst studies that report on objective outcomes after non-surgical treatment are currently limited.^23,24^ Furthermore, although feeding and growth are suggested to be important issues in patients with RS due to the high prevalence and serious complications when left untreated, feeding and growth outcomes following (non-surgical) treatment are scarce. Therefore, we feel that these functionalities should be taken into account when describing treatment outcomes. The aim of this study was to reflect upon our non-surgical respiratory treatment protocol by prospectively evaluating clinical outcomes regarding airway, feeding, and growth during the first year of life in patients with RS.

## Materials and Methods

A prospective longitudinal study was conducted on children diagnosed with RS who were treated between 2011 and 2021 in the Sophia Children's Hospital, Rotterdam, the Netherlands. The study was approved by the by the medical ethical committee of the Erasmus Medical Center (MEC-2012-048 & MEC-2017-126). Patients were considered eligible for inclusion if they were (1) diagnosed with both isolated and non-isolated RS, defined as the presence of clinical mandibular hypoplasia, clinical glossoptosis, and the presence of UAO, objectified by a polysomnography (PSG) and/or pulse oximetry; (2) treated during their first 3 months of life in our hospital; (3) had a follow-up in our center up to at least 1 year of age; and (4) both parents of patients with RS had given written informed consent. Patients were excluded from further analysis if they received any surgical treatment like placement of a tracheal cannula or mandibular distraction osteogenesis.

### Outcome Measures

The following demographic data were gathered: date of birth, gestational age (GA) at birth, sex, presence of a cleft palate, presence of a genetic syndrome (clinically or genetically confirmed), and the presence of additional comorbidities. All level 1 PSG's that were performed throughout infancy in our center were gathered. With level 1 PSG, the following parameters were assessed: nasal airflow, chest and abdominal movement, capillary blood gas samples (eg, CO_2_, HCO_3_-, Base Excess), SpO_2_ using pulse oximetry, transcutaneous CO_2_, a full-night video recording, an electrocardiogram (ECG), and an electroencephalogram (EEG). PSG's at time of initiation of treatment (PSG1), at time of discontinuation of treatment (PSG2), and at the age of 1 year (PSG3) were included for final analysis. In case of the occurrence of a cleft palate, the latter was often performed with a removable custom-made orthodontic plate that simulates palatal closure, around the age of 1 year, prior to palatal closure.

All PSG's were analyzed using Shell+ BrainRT Software (Suite Version 2.0; O.S.G. Rumst, Belgium) and scored according to the guidelines of the American Academy of Sleep Medicine.^25^ Based on the combination of clinical observation, PSG characteristics, and nocturnal capillary blood gas values, severity of UAO was assessed and management strategies were settled by the multidisciplinary team. PSG characteristics included obstructive apnea hypopnea index (oAHI) values, mean oxygen saturation, maximal desaturation (oxygen saturation nadir), oxygen desaturation index >4% (ODI > 4%). Nocturnal capillary blood gas values included pCO_2_, HCO_3_-, and Base Excess (BE).

Based on the ethical principle to start with the least invasive but most effective intervention, neonates with mild-to moderate UAO were indicated for non-surgical management. Non-surgical treatment options included positional therapy (prone and/or side positioning) (PT) or respiratory support, including supplemental oxygen, placement of a nasopharyngeal airway (NPA) with or without supplemental oxygen, non-invasive ventilation (NIV), continuous positive airway pressure (CPAP), and high flow nasal cannula (HFNC). Patients who were treated with PT were sent home (after the PSG) with home pulse oximetry monitoring. In case an NPA was used, patients remained admitted throughout the entire period of treatment. The largest size of the NPA (Smiths Medical Siliconised PVC, Oral/Nasal Uncuffed Tracheal Tube ®) that could be inserted without resistance was used. Via fiberoptic laryngoscopy, at first insertion, depth was checked and adjusted when needed.”

If needed, supplemental oxygen, CPAP or NIV was initiated in our hospital. Patients were discharged if they were clinically stable and parents were trained to safely carry out the treatment (oxygen, CPAP or NIV). In the Netherlands, at the time of our study, it was not possible to administer HFNC at home. Throughout treatment with HFNC patients remained admitted.

In patients treated with respiratory support, severity of UAO was re-evaluated by clinical assessment and a PSG after 6 weeks-3 months and, if indicated, a change in treatment was considered. In patients treated with PT, reevaluation by a PSG was only performed on clinical indication. Neonates born with severe UAO were intubated directly after birth. Based on prenatal findings (if present), clinical assessment, severity of respiratory distress (capillary blood gas values and if performed an oAHI and/or a naso-endoscopy), and/or intubation conditions, a decision was made to either place a tracheostomy tube or to leave the patient intubated and, if possible, extubate on trial after 1 week.

Data on type and duration of initial airway management, subsequent airway management, and subsequent airway-related interventions was collected. Information regarding airway related severe adverse events or complications was gathered. The age at time of cleft palate closure was reported. Patients who were only shortly (<3days) treated with respiratory support after birth due to transitional problems of the newborn (e.g. wet lung) were included in the PT-group.

Feeding outcomes were classified according to the classification system of Caron et al., based on the ability to be orally fed.^26^ “No-mild” FD were diagnosed if patients were able to be fully orally fed. FD were diagnosed as “moderate” if patients were able to be orally fed but required tube feeding for adequate intake. FD were diagnosed as “severe” if patients were fully tube feeding-dependent. If present, type and duration of tube feeding were reported. Judged by the specialized speech and language therapist (SSLT), dietician and clinician (multidisciplinary team), patients were fed by mouth whenpossible. In our center, we aim to emulate an individualized approach. Accordingly, based on the outcomes of several examinations of the multidisciplinary team (including an SSLT and dietician), the probability of long-term FD (especially swallowing difficulties) is estimated. Based on that estimation, a decision is made whether or not to place a PEG-tube.

Individual weights and heights were gathered and evaluated using growth charts. Heights and weights were expressed in standard deviation scores (SDS) for weight-for-age (WFA), and height-for-age (HFA), compared to the published standards of the Dutch reference population. Acute malnutrition was defined as WFA < −2SDS. Chronic malnutrition was defined as HFA < −2SDS from the age of 1 year onwards.^27,28^

### Statistical Analysis

Statistical analysis was performed using SPSS statistics version 28.0 for Windows (IBM Corp., Armonk, N.Y., USA). For counts, descriptive statistics were illustrated as numbers with percentages. Numeric data were reported as means with standard deviations (±SD) for normally distributed data or as medians with interquartile ranges (IQR) for non-normally distributed data. To examine outcomes prior and after treatment within individual patients, a paired T-test was performed for normally distributed data or a Wilcoxon signed rank test for non-normally distributed data. To compare patients treated with PT versus patients treated with respiratory support and to compare isolated patients with RS versus non-isolated patients with RS, an independent T-test was performed for normally distributed data or a Mann Whitney U-test for non-normally distributed data. P-values lower than 0.05 were considered as statistically significant.

## Results

A total of 64 patients were diagnosed with RS between 2011 and 2021 in our center and considered for inclusion. Twelve patients were excluded because they presented after their first 3 months of life in our hospital, of whom six were surgically treated (all tracheostomy), one received CPAP, two required an NPA and three were treated with positional therapy. Four patients were deceased before informed consent could be obtained and five parents did not give informed consent. Parents of 43 children gave informed consent and their children were therefore included in the longitudinal study. Two patients were excluded from further analysis because they were referred to another center during the first year of life, of which one was treated with positional therapy and one was treated with an NPA. Another five patients received surgical treatment (tracheostomy n = 5) during their first year of life and were subsequently excluded for further analysis.

**Figure 1.**
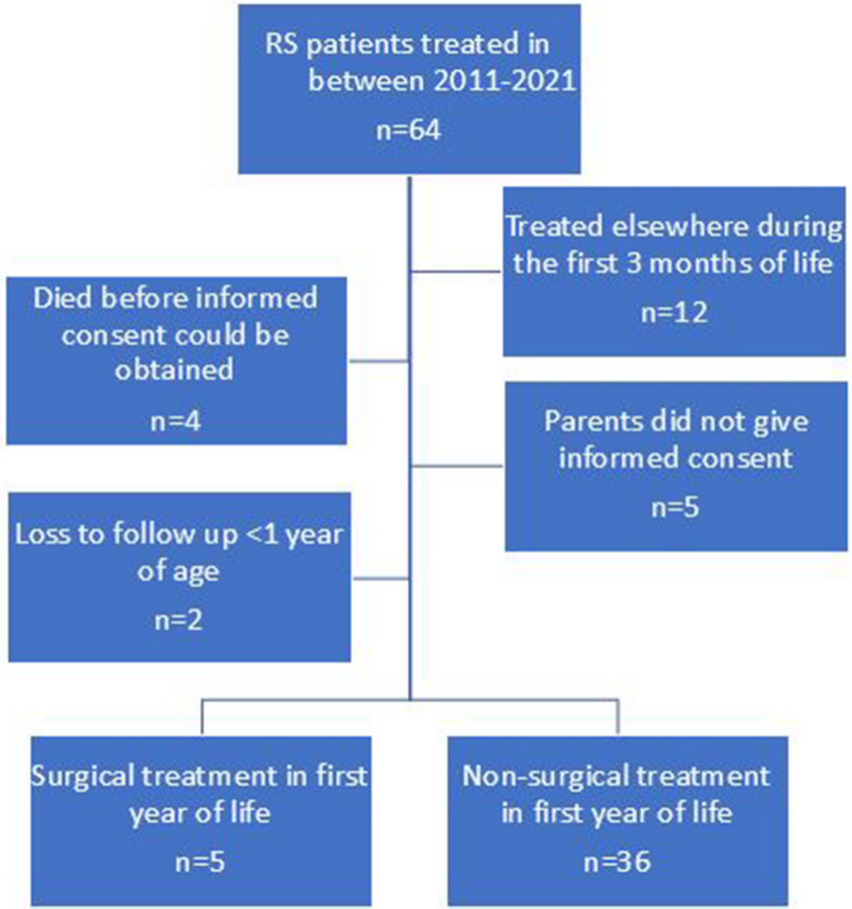
Flow chart of inclusion and exclusion of patients.

Abbreviations: RS, robin sequence; EMC, Erasmus Medical Center.

A total of 36 patients was included in final analysis (Figure 1). Twenty-three (64%) out of 36 patients were male. Thirty (83%) patients presented with a cleft palate, whilst 22 (61%) patients presented with non-isolated RS of whom 10 presented with a syndromic diagnosis (Stickler n = 6; 22q11deletion syndrome n = 1; 19q13 microdeletion syndrome n = 1; Anderson Tawil syndrome n = 1, Trisomy 9 n = 1). The other 12 patients presented with additional comorbidities (RS-plus), including cardiac abnormalities, neurological dysregulation, limb abnormalities, and other craniofacial abnormalities (eg, ear abnormalities, skin tags, ocular abnormalities). One patient died at the age of 5 months at home due to respiratory problems.

### Respiratory Outcomes

**Figure 2.**
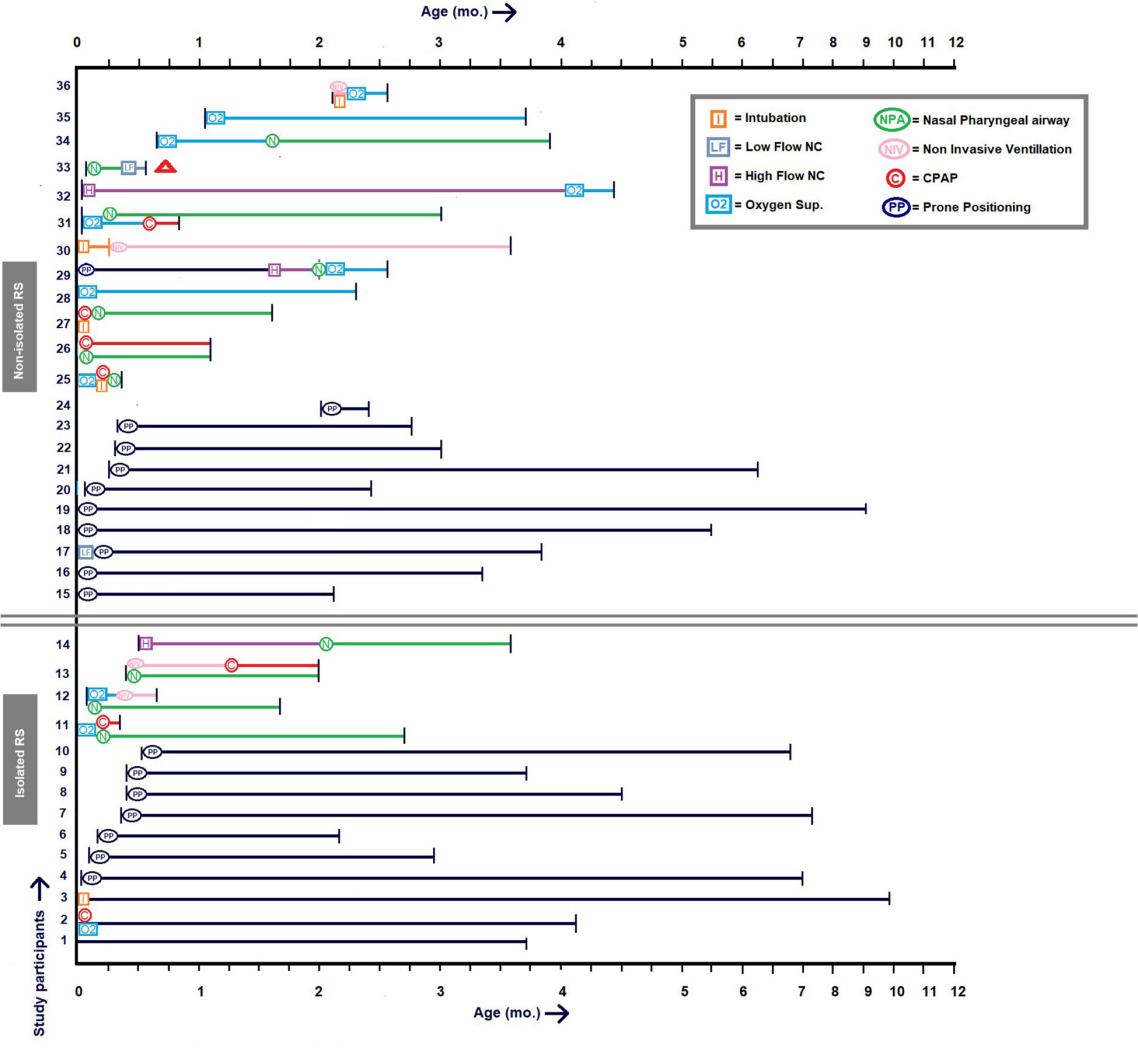
Overview of respiratory management during the first year of life.

Twenty out of 36 patients were treated with positional therapy, of whom three patients shortly received respiratory support during their first 2-4 days of life because of transitional respiratory problems of the newborn. The other 16 patients (non-isolated RS n = 12) were treated with respiratory support, of whom 11 patients required an NPA with subsequently other types of respiratory support prior (n = 5), during (n = 5), or after (n = 1) NPA treatment. The latter required low flow supplemental oxygen after NPA for an additional five days. No patients were discharged with respiratory support. Four out of 16 patients were intubated for a maximum of 8 days, and subsequently received other types of respiratory support. Fifteen patients started with treatment at their day of birth. Median age at time of start treatment was 1.5 (IQR 0.0 - 12.0) days. Median duration of all treatment was 3.1 (IQR 2.2 - 4.3) months. Median duration of respiratory support was 2.4 (IQR 0.6 - 3.1) months, with a median duration of 1.5 (IQR 0.3 - 2.3) months for NPA specifically. Median duration of PT was 3.8 (IQR 2.8 - 6.6) months. Individual treatment pathways are depicted in Figure 2.

Age is represented in months (mo.). Abbreviations: NC, nasal cannula; CPAP, continuous positive airway pressure.

**Figure 3.**
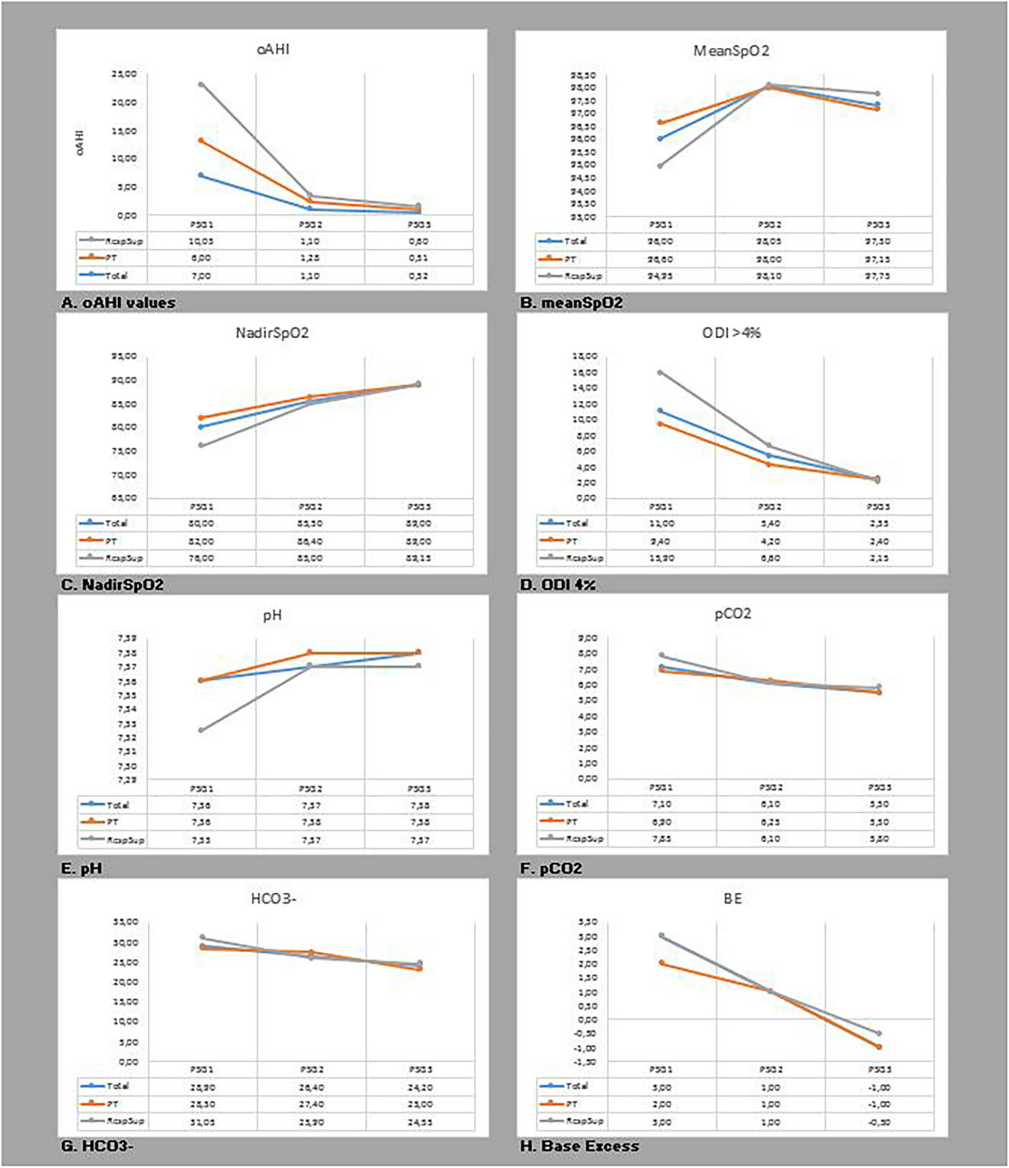
A-H - Change of oAHI and capillary blood gas values over time.

Abbreviations: oAHI, obstructive apnea hypopnea index; Mean SpO2, mean oxygen saturation in blood (%); Nadir SpO2, lowest oxygen saturation in blood (%); ODI, Oxygen Desaturation Index; pH, acidity of blood; pCO2, carbon dioxide concentration in blood; HCO3-, actual bicarbonate concentration in blood; BE, Base Excess.

At time of start treatment, median oAHI of all patients (n = 31) was 7.0 (IQR 4.3-12.2). At time of stop treatment (n = 20), median oAHI was 1.1 (IQR 0.5-2.7). At the age of 1 year (n = 30), median oAHI was 0.5 (IQR 0.1-0.9). Median oAHI values were significantly different at each time interval, respectively PSG1 vs PSG2 *P* < .001; PSG1 vs PSG3 *P* < .001; PSG2 vs PSG3 *P* = .006. All capillary blood gas values improved over time and returned to a value within the range of the normal reference population at time of PSG3, except for HCO3- (Table 1 and Figure 3).

All variables are expressed in median.

**Table 1.** oAHI Values and Capillary Blood Gas Values Over Time.

	PSG 1 vs 2 n = 16	*P*-value	PSG 1 vs 3 n = 28	*P*-value	PSG 2 vs 3 n = 15	*P*-value
oAHI	8.24 (4.50-15.61) vs 1.35 (0.72-2.99)	<.001	6.42(4.06-10.47) vs 0.49 (0.12-0.92)	<.001	1.28 (0.60-2.82) vs 0.50 (0.23-0.90)	.006
MeanSpO2	96.00 (94.33-96.90) vs 98.05(97.05-98.45)	.007	96.15 (94.30-97.15) vs 97.35 (96.00-98.00)	.056	98.00 (97.00-98.30) vs 97.30 (96.00-98.30)	.280
O2nadir	78.00 (71.50-82.30) vs 85.50 (83.25-87.00)	.002	80.00 (76.00-83.00) vs 89.00 (86.03-92.75)	<.001	85.00 (79.00-87.00) vs 87.00 (85.00-93.00)	.118
ODI4%	14.00 (5.30-28.00) vs 5.40 (2.30-10.40)^a^	.078	9.70 (5.78-22.68) vs 2.05 (1.00-8.48)	<.001	5.40 (2.30-10.40) vs 4.00 (1.00-9.20)	.363
pH	7.36 (7.32-7.36) vs 7.37 (7.36-7.38)^a^	.059	7.36 (7.32-7.36) vs 7.38 (7.36-7.39)	<.001	7.37 (7.36-7.38) vs 7.38 (7.36-7.39)^c^	.725
pCO2	7.50 (6.90-8.00) vs 6.10 (5.70-6.80)^a^	.002	7.15 (6.63-8.00) vs 5.50 (5.10-5.88)	<.001	6.25 (5.78-6.90) vs 5.80 (5.48-6.03)^c^	.059
HCO3-	30.40 (28.00-32.00) vs 26.40 (24.80-28.10)^a^	.005	28.90 (27.70-31.40) vs 23.90 (22.70-25.40)^b^	<.001	27.40 (24.95-28.40) vs 24.55 (23.85-25.60)^c^	.009
BE	3.00 (1.00-5.00) vs 1.00 (0.00-2.00)^a^	.063	3.00 (1.00-4.00) vs −1.00 (−2.00-0.00)	<.001	1.00 (0.00-2.25) vs −0.50 (−2.00-1.00)^c^	.019

Abbreviations: PSG, polysomnography; oAHI, obstructive apnea hypopnea index; Mean SpO2, mean oxygen saturation in blood(%); Nadir SpO2, lowest oxygen saturation in blood(%); ODI, Oxygen Desaturation Index; pH, acidity of blood; pCO2, carbon dioxide concentration in blood HCO3-, actual bicarbonate concentration in blood; BE, Base Excess.

All variables are expressed in median (interquartile range (IQR)).

^a^ 15 patients involved in final analysis.

^b^ 27 patients involved in final analysis.

^c^ 14 patients involved in final analysis.

Prior to treatment (PSG1), significant differences were found in mean oxygen saturation and oxygen saturation nadir between patients treated with PT and patients treated with respiratory support (*P* = .048; *P* = .005, respectively). No significant differences were found in age, oAHI values, and values of ODI, pH, pCO2, HCO3-, and BE between both groups. At time of stop treatment (PSG2), no significant differences were found between both groups in oAHI values or any of the capillary blood gas values. At the age of 1 year (PSG3), significant differences were found between isolated and non-isolated patients with RS regarding mean oxygen saturation, oxygen saturation nadir, and ODI > 4% (*P* = .028; *P* = .002; *P* = .017, respectively). No significant differences were found for: age, oAHI values, or any of the capillary blood gas values (Table 2).

**Table 2.** Polysomnographic Results Prior to Treatment, After Treatment and at the Age of 1 Year.

	PSG1		PSG2		PSG 3	
	Positional therapy (n = 19)	Respiratory support (n = 12)	Positional therapy (n = 7)	Respiratory support (n = 13)	Positional therapy (n = 18)	Respiratory support (n = 12)
Age (days)	12,.00 (7.00-18.00)	26.00 (9.00-71.50)	124.00 (117.00-198.00)^a^	106.00 (53.50-126.00)^a^	295.00 (280.00-429.25)	370.50 (326.50-431.75)
oAHI	6.00 (4.00-10.08)	10.03 (6.07-17.08)	1.28 (0.60-1.53)	1.10 (0.42-3.14)	0.51 (0.12-0.91)	0.60 (0.11-2.51)
Mean SpO2 (%)	96.60 (95.50-97.20)^a^	94.95 (93.63-96.30)^a^	98.00 (96.50-98.50)	98.10 (96.50-98.50)	97.15 (95.75-98.00)	97.75 (96.00-98.30)
Nadir SpO2 (%)	82.00 (78.00-83.20)^a^	76.00 (68.85-78.75)^a^	86.40 (79.00-89.20)	85.00 (81.00-87.00)	89.00 (86.40-91.00)	89.15 (80.25-93.75)
ÒDI < 4%	9.40 (5.20-22.80)	15.90 (8.38-33.03)	4.20 (3.05-7.48)^b^	6.60 (2.70-14.00)	2.40 (1.10-7.23)	2.15 (0.63-9.55)
pH	7.36 (7.35-7.37)	7.33 (7.31-7.36)	7.38 (7.34-7.43)^b^	7.37 (7.36-7.38)	7.38 (7.36-7.40)	7.37 (7.36-7.39)
pCO2	6.90 (6.60-7.30)	7.85 (6.50-8.20)	6.25 (5.28-6.90)^b^	6.10 (5.80-7.05)	5.50 (5.10-5.73)	5.80 (5.28-6.08)
HCO3-	28.30 (27.70-30.40)	31.05 (27.55-32.45)	27.40 (24.75-28.13)^b^	25.90 (25.00-29.50)	23.00 (22.70-24.90)^c^	24.55 (23.75-25.83)
BE	2.00 (1.00-4.00)	3.00 (1.25-4.75)	1.00 (0.75-2.00)^b^	1.00 (-0.50-3.00)	−1.00 (−2.00-0.75)	−0.50 (−1.00-0.75)
	Isolated RS n = 14	Non-isolated RS n = 17	Isolated RS n = 7	Non-isolated RS n = 13	Isolated RS n = 14	Non-isolated RS n = 16
Age (days)	12.00 (7.50-15.25)	20.00 (7.50-59.50)	108.00 (61.00-124.00)	117.00 (54.50-151.00)	303.50 (280.00-431.00)	349.50 (293.50-431.75)
oAHI	6.29 (2.38-10.21)	7.56 (5.25-15.35)	1.08 (0.44-1.53)	1.28 (0.30-3.14)	0.23 (0.11-0.83)	0.75 (0.35-1.90)
Mean SpO2 (%)	96.45 (95.20-97.23)	96.00 (94.00-97.10)	98.10 (96.50-98.60)	98.00 (96.40-98.45)	97.90^a^ (97.00-98.33)	96.00^a^ (95.25-97.88)
Nadir SpO2 (%)	78.50 (72.50-82.53)	80.00 (74.00-83.00)	86.00 (79.00-97.00)	84.00 (81.00-87.50)	91.50^a^ (88.50-93.25)	86.50^a^ (80.25-89.00)
ÒDI < 4%	11.70 (4.75-23.73)	11.00 (8.25-26.20)	4.00 (2.00-5.40)	6.95 (3.80-15.30)	1.50^a^ (0.28-3.85)	5.55^a^ (1.80-11.38)
pH	7.36 (7.35-7.37)	7.34 (7.32-7.36)	7.37^b^ (7.36-7.38)	7.38 (7.35-7.39)	7.37 (7.36-7.39)	7.38 (7.37-7.39)
pCO2	7.00 (6.48-7.78)	7.20 (6.70-8.05)	6.05^b^ (5.70-6.58)	6.40 (5.75-7.20)	5.45 (5.10-5.83)	5.60 (5.13-6.05)
HCO3-	29.30 (27.30-31.33)	28.90 (27.85-32.00)	26.40^b^ (24.65-28.10)	26.40 (25.00-29.50)	23.45 (22.70-24.43)	25.00^c^ (22.90-25.90)
BE	2.50 (1.00-4.00)	3.00 (1.00-4.00)	0.50^b^ (−0.25-2.00)	1.00 (0.00-3.00)	−1.00 (−2.00-−1.00)	−0.50 (−2.00-1.75)

Abbreviations: PSG, polysomnography; oAHI, obstructive apnea hypopnea index; Mean SpO2, mean oxygen saturation in blood (%); Nadir SpO2, lowest oxygen saturation in blood (%); ODI, Oxygen Desaturation Index; pH, acidity of blood; pCO2, carbon dioxide concentration in blood HCO3-, actual bicarbonate concentration in blood; BE, Base Excess; isolated RS, patients with isolated Robin Sequence; non-isolated RS, patients with non-isolated Robin Sequence.

Respiratory support, including supplemental oxygen (O2), placement of a nasopharyngeal airway (NPA), non-invasive ventilation (NIV), continuous positive airway pressure (CPAP), and low flow nasal cannula (LFNC) or high flow nasal cannula (HFNC) with O2.

All variables are expressed in median (interquartile range (IQR).

^a^ Significantly different (*P* < .005).

^b^ Of only 6 patients data was available on capillary blood gas values and therefore included in analysis.

^c^ In one patient HCO3- level was not available and therefore a total of 17 patients wasincluded in final analysis.

### Feeding Outcomes

A total of 31 (86%) patients required tube feeding during their first year of life. The other 5 patients, all treated with PT, were able to be fully orally fed (no-mild FD). All 31 patients were initially fed via a nasogastric (NG-) tube, of whom 26 were solely fed by an NG-tube. Five patients required additional feeding mechanisms following the NG-tube: four patients received a PEG-tube, in whom one the PEG was replaced by a jejunostomy. In the fifth patient, the NG-tube was replaced by a transpyloric tube. During the first year of life, 23 out of 31 patients with FD demonstrated improved feeding abilities whilst the other eight patients did not improve at all. All five patients with no-mild FD remained able to be fully orally fed. At 1 year of age, 27 patients were able to be fully orally fed (no-mild FD) and 9 patients still had FD (moderate FD n = 3; severe FD n = 6), of whom 7 were treated with respiratory support. Eight out of 9 patients who had persisting FD at the age of 1 year, presented with non-isolated RS. Thirty-five patients were guided by an SSLT (inside our center n = 28; outside our center n = 7). Sixteen out of 36 patients (44%) were consulted by a dietician. Nineteen patients (53%) received feeding with extra calories and/or extra protein.

### Growth Outcomes

Four patients were born prematurely (GA, range 33+1 - 36+5 weeks, median birthweight 2002 (IQR 1825 - 2896) gram). Thirty-two patients were born term (median GA 39+6 (IQR: 38+2 - 40+3 weeks), median birthweight 3249 (IQR 3051 - 3697) gram). At time of start treatment, corrected for gestational age and sex, median SDS WFA was −0.40 (IQR −1.16 - 0.26) and was significantly lower in patients who received respiratory support compared to those treated with PT (*P* = .003). Four patients presented with acute malnutrition (WFA < −2SD) at time of start treatment. During treatment, the median growth rate was 153 (IQR 121 - 183) gram per week. At time of stop treatment, median SDS WFA was −0.33 (IQR −1.56 - 0.17). Seven patients (all non-isolated RS) were still malnourished (WFA < −2SDS). The median increase of weight after treatment until the age of 1 year was 83 (IQR 65 - 101) gram per week. At the age of 1 year, the median SDS for WFA was −1.03 (IQR −1.66 - −0.14) and median SDS for HFA was −0.71 (IQR −1.37 - 0.26). At 1 year follow-up, malnutrition was present in 7 (19%) patients (non-isolated RS n = 6). Growth outcomes are presented in Table 3.

**Table 3.** Growth Outcomes in Isolated Patients with RS vs. Non-Isolated Patients with RS.

Group Variable	Isolated RS n = 14	Non-isolated RS n = 22	*P*-value	Positional therapy n = 20	Respiratory support n = 16	*P*-value	Total n = 36
Birth weight (kg)	3.252 (3.074-3.489)	3.140 (2.333-3.613)	.267	3.302 (3.057-3.897)	3.113 (2.407-3.359)	.053	3.168 (2.980-3.543)
Start treatment	n = 14	n = 22		n = 20	n = 16		n = 36
SDS WFA	−0.286 (−0.929-0.194)	−0.495 (−1.381-0.383)	.642	−0.044 (−0.471-0.792)	−0.915 (−1.608-0.163)	.003	−0.398 (−1.159-0.264)
Stop treatment	n = 13	n = 22		n = 19	n = 16		n = 35
SDS WFA	−1.123 (−0.610-0.192)	−1.027 (−2.252-0.167)	.057	−0.297 (−1.516-0.147)	−0.802 (−2.399-0.251)	.806	−0.330 (−1.555-0.174)
Growth (g) per week	163.000 (137.150-192.500)	144.450 (110.150-172.700)	.229	142.500 (125.000-163.200)	168.550 (113.850-207.925)	.182	152.700 (120.600-182.600)
At age 1 y	n = 12	n = 20		n = 18	n = 14		n = 32
Weight (kg)	8.800 (8.535-9.425)	8.480 (7.543-9.601)	.408	8.770 (8.053-9.004)	8.900 (7.846-10.314)	.722	8.770 (7.831-9.500)
SDS WFA	−0.883 (−1.273-0.065)	−1.165 (−2.628-−0.289)	.107	−1.109 (−1.555-−0.128)	−0.980 (−2.475-0.067)	.866	−1.030 (−1.669-−0.141)
Growth (g) per week^a^	80.100 (58.775-94.150)	85.400 (66.700-111.125)	.146	76.550 (61.475-95.875)	94.050 (72.900-114.050)	.091	83.150 (65.200-101.050)
Length (cm)^b^	75.050 (71.825-77.200)	73.000 (69.575-75.750)	.397	74.100 (71.050-77.400)	73.000 (70.150-76.500)	.869	73.550 (71.075-77.000)
SDS HFA^c^	−0.359 (−1.074-0.264)	−0.854 (−1.482-0.331)	.350	−0.604 (−1.244-0.282)	−0.946 (−1.450-0.016)	.432	−0.714 (−1.377-0.264)

Abbreviations: SDS, Standard Deviation Score; WFA, Weight for Age; HFA, Height for Age; isolated RS, patients with isolated Robin Sequence; non-isolated RS, patients with non-isolated Robin Sequence.

All variables are expressed in median (interquartile range (IQR).

^a^ Growth per week after treatment until age of 1 year.

^b^ In only 10 isolated patients with RS and 20 non-isolated patients with RS, length at the age of 1 year was available, leading to a total of 30 patients with RS included for analysis.

^c^ In only 17 patients with RS treated with PP and 13 patients with RS treated with respiratory support, length at the age of 1 year was available, leading to a total of 30 patients with RS included for analysis.

## Discussion

In this prospective longitudinal study of 36 mildly-to moderately affected patients with RS who were non-surgically treated, the respiratory management in relation to feeding disorders and growth was evaluated. Treatment consisted either of positional therapy (PT) or respiratory support. This study found a near complete improvement of UAO in both treated groups during the first year of life. Median duration of treatment of the respiratory support group was 2.4 months and in the PT group 3.8 months. Although treatment of UAO was relatively short, FD persisted in 25% of the patients whilst 20% of the patients were still diagnosed with malnutrition at the age of 1 year.

In our center, therapeutic decisions in the management of RS were based on clinical symptoms of UAO and results from the polysomnography (PSG), which is the gold standard to diagnose obstructive sleep apnea (OSA).^2,22,29^ Nonetheless, defining severity of OSA, especially in young children, seems to be challenging.^23,30^ Until now, over 30 definitions have been used in literature to define presence and severity of OSA in children with RS.^23^ Solely relying on PSG values may be misleading.^30^ Healthy young neonates and infants are predisposed to UAO and hypoxemia due to an increased upper airway and chest wall compliance and an immature breathing pattern and therefore may also present with raised (o)AHI-values.^30–35^ Conversely, Tan & Kaditis recently stated that some children may suffer from sleep disordered breathing, but can have a (seemingly) normal PSG.^36^ Moreover, since paradoxal breathing is very common in infants during REM sleep, differentiating between central and obstructive hypopneas can be challenging, resulting in an incorrectly high (o)AHI-value.^30^ Lastly, timing of a PSG may be essential due to the fact that during infancy, (o)AHI-values, ODI-values, and sleep architecture variables evolve over time.^30^ Due to the difficulties in interpreting a PSG in young infants, together with the limited reliability of the use of oAHI-values only, other clinical parameters and laboratory values should be taken into account to decide about treatment.

Previous studies suggested the usefulness of nocturnal pulse oximetry to assess severity of OSA in infants that present with symptoms of respiratory distress, especially when a PSG is not feasible to perform.^37,38^ Prior to treatment, our study found significant lower mean oxygen saturations and more often a lower deepest oxygen saturation drop in patients who required respiratory support compared to those who were treated with PT, reflecting the preference to start with PT in the patients with milder consequences of obstructive breathing. This is comparable to previous findings in a study of 55 infants with RS, in which a higher clinical severity score (according to Cole's classification) was associated with a significantly higher odds to have lower mean oxygen saturation and a lower oxygen saturation nadir.^38^ Nonetheless, sensitivity of pulse oximetry for OSA in patients with RS is still lower compared to PSG outcomes.^39^ Since desaturations related to central apnea are very common in (healthy) neonates, differentiating central events from obstructive events is essential in judging the severity of OSA. However, pulse oximetry data only provides information on duration and depth of desaturation, but not of the initial cause, which may lead to an inaccurate interpretation of the severity of respiratory distress.^23,30^

Besides pulse oximetry data, capillary blood gas values can also provide quantifiable characteristics for the presence and severity of respiratory distress in patients with RS.^40–42^ In combination with clinical signs of OSA, hypercapnia (PaCO_2_ > 50 mmHg), that is present for more than 25% of total sleep time, is indicative for the presence of OSA.^43^ Kwann et al. suggested that elevated capillary blood gas values in the first weeks of life are associated with the need for airway intervention in patients with RS.^42^ Accordingly, although not significant, we found a lower value for pH and higher levels for pCO2, HCO3-, and BE in patients treated with respiratory support compared to those treated with PT.

Concerning respiratory support in this study, different respiratory modalities were used, of which the NPA is considered the first choice of treatment. The median duration of NPA treatment was 1.5 months, which seems to be comparable to other studies that inserted an NPA.^44–47^ In our study, smaller groups of patients were successfully treated with NIV, CPAP, LFNC or HNFC. The median duration of total treatment of the respiratory support group was 2.4 months and started at a mean age of 8 days. This suggests that in some patients there seems to be a ‘Honeymoon period”: during the first days in life, patients seem to do quite well and signs of respiratory distress are not experienced or clinically of such mild severity that they are not recognized by parents or clinicians. However, after a (short) postnatal period of respiratory stability, changes in respiratory status occur and UAO is developed. A recent study by Wilson et al. already reported on the potential for late presentation of respiratory distress in patients with RS, with an onset up to 51 days.^48^ Although it is possible that UAO was already present in the early neonatal period, it seems that respiratory distress can develop progressively during the first weeks of life. Noteworthy, four out of 16 patients in the respiratory support group were intubated for a maximum of 8 days. Thereafter, they were extubated on trial and switched to other types of respiratory treatment.

Although not required for diagnosis, a high prevalence of FD (83%) in patients with RS was found by this study. Besides underlying diagnosis,^49–52^ FD are closely associated with the presence and severity of UAO.^49,52–54^ At time of first presentation, FD were present in all of our patients who required additional respiratory support, whilst 75% of the patients who were treated with PT required additional tube feeding. These findings are in line with the results of a previous study in which a significant difference was found in the need for tube feeding between patients with RS with mild-moderate UAO compared to those with severe UAO.^53^

Several studies suggest that treating UAO should result in an improvement in feeding abilities.^45,52,55^ In our study, however, 25% of the patients still required tube feeding at the age of 1 year, despite improvement of respiratory symptoms. Remarkably, all (except one) were patients with non-isolated RS. These findings support the hypothesis that FD are often multifactorial of cause (eg, underlying neurological deficits, swallowing problems) and that resolution of UAO not necessarily implies that FD will be completely dissolved.

Along with FD, patients with RS are at an increased risk of enduring malnutrition and impaired growth.^7,54,56^ At time of start treatment, our patients with RS presented with a lower SDS WFA compared to their healthy peers. In patients treated with respiratory support, even a significantly lower weight was found compared to patients treated with positional therapy (*P* = .003). Interestingly, although SDS WFA at stop treatment and at the age of 1 year remained lower in patients treated with respiratory support compared to patients treated with PT, this difference was not significant. An explanation could be that improvement or resolution of UAO in the more severely affected patients results in a greater decrease of energy requirements, subsequently leading to an acceleration of weight gain.^57–59^ Previous studies have found that sufficient treatment of UAO in patients with RS results in catch up growth after non-surgical treatment.^53,54^ In this study, however, we found low SDS for WFA and HFA compared to the healthy pediatric population at the age of 1 year, which indicates a notable delay in growth, even after treatment of the UAO.

Remarkably, we did not notice respiratory problems as a cause of impaired growth at the age of 1 year. Even more, this study found that 6 out of nine 9 patients that remained tube feeding-dependent at the age of 1 year were malnourished (SDS WFA < 2 SDS), despite (additional) tube feeding. Findings remain contrasting whether there is an inherit growth deficit in patients with RS or that the low SDS WFA and HFA are caused by underlying comorbidities, insufficient intake secondary to the FD, or that other factors might interfere with appropriate growth. In contrast to what many would assume, we did not find significant differences in both SDS WFA and SDS HFA between isolated and non-isolated patients with RS.

Alarmingly, we found that the median SDS for WFA declined 0.63 SDS during the first year of life and that the median SDS for HFA at the age of 1 year was low (−0.71 SDS). The findings that in our study only a small majority of patients (53%) received feeding with extra calories and/or extra protein and that in less than half of our patients (44%) a dietician was consulted were also worrisome. Given the high prevalence of persisting FD and impaired growth, a multidisciplinary approach and the implementation of routine nutritional assessment on standardized time points is needed in the management of patients with RS.

Prospective longitudinal studies that focus on the FD and long-term growth trajectories are required to delineate characteristics that are associated with impaired feeding and growth outcomes. Comparing functional outcomes of different non-surgical treatment modalities would aid in optimizing management strategies. Moreover, whilst our protocol is predominantly based on a non-surgical approach, some studies advocate for early surgical intervention since postponed surgery is claimed to be less effective in cases that eventually require surgery^60–62^. However, when reviewing our results we can conclude that a large number of patients do not require surgery for resolution of their respiratory distress. When surgery is done on these patients, the successful outcome may be unjustly attributed to the surgical intervention. Therefore, it would be interesting to compare our outcomes to the outcomes of centers that follow a more surgically-based protocol in relation with long-term complications associated with (insufficiently treated) UAO. Furthermore, by showing our findings, we attempt to encourage other studies to continue in this field of research to improve knowledge.

Some limitations have to be addressed. The small sample size and the heterogeneity of the (non-isolated) patient population might influence the generalizability of our results. Since not all eligible patients were included in the current study, results might have been influenced by selection bias. Furthermore, by focusing on non-surgically treated patients, our cohort potentially consists of patients with RS with a milder phenotype, making our results less applicable for patients who for example require tracheostomy in early infancy. However, our study shows that even in patients with mild-moderate UAO, severe problems regarding growth and feeding occur. Finally, information bias poses a limitation since some patients were also treated, besides they received treatment in our center, in other centers. This may have resulted in missing data or loss of information. Besides, not all information (eg, information on clinical symptoms and timing of stop treatment, especially of positional therapy) was consistently reported and needed to be assessed from data available in the electronic patient file.

## Conclusions

Non-surgical respiratory treatment in patients with RS resulted in an improvement of clinical symptoms, PSG characteristics, and capillary blood gas values to near normal during the first year of life. These patients with RS, however, often experienced feeding difficulties and endured impaired weight gain and height growth compared to their healthy peers up to 1 year of age. The persistent high prevalence of FD at the age of 1 year and the ongoing decline in SDS WFA at 1 year follow-up is alarming and indicates the urgency of a multidisciplinary approach and the implementation of routine nutritional assessment to support optimal growth. Future studies should focus on FD and growth in relation with long-term outcomes (>1 year) in these patients.

## References

[1] Paes EC van Nunen DP Basart H , et al. Birth prevalence of Robin sequence in The Netherlands from 2000-2010: a retrospective population-based study in a large Dutch cohort and review of the literature. Am J Med Genet A. 2015;167A(9):1972-1982. 25994858 10.1002/ajmg.a.37150

[2] Breugem CC Evans KN Poets CF , et al. Best practices for the diagnosis and evaluation of infants with robin sequence: a clinical consensus report. JAMA Pediatr. 2016;170(9):894-902. 27429161 10.1001/jamapediatrics.2016.0796

[3] Caouette-Laberge L Bayet B Larocque Y . The Pierre Robin sequence: review of 125 cases and evolution of treatment modalities. Plast Reconstr Surg. 1994;93(5):934-942. 8134485

[4] Xu JX Kilpatrick N Baker NL Penington A Farlie PG Tan TY . Clinical and molecular characterisation of children with Pierre Robin sequence and additional anomalies. Mol Syndromol. 2016;7(6):322-328. 27920635 10.1159/000449115 PMC5131331

[5] Izumi K Konczal LL Mitchell AL Jones MC . Underlying genetic diagnosis of Pierre Robin sequence: retrospective chart review at two children's hospitals and a systematic literature review. J Pediatr. 2012;160(4):645-650.e2. 22048048 10.1016/j.jpeds.2011.09.021

[6] Cohen MM Jr . Robin sequences and complexes: causal heterogeneity and pathogenetic/phenotypic variability. Am J Med Genet. 1999;84(4):311-315. 10340643

[7] Paes EC de Vries IAC Penris WM , et al. Growth and prevalence of feeding difficulties in children with Robin sequence: a retrospective cohort study. Clin Oral Investig. 2017;21(6):2063-2076. 10.1007/s00784-016-1996-8 PMC5487830 27868158

[8] Cooper-Brown L Copeland S Dailey S , et al. Feeding and swallowing dysfunction in genetic syndromes. Dev Disabil Res Rev. 2008;14(2):147-157. 18646013 10.1002/ddrr.19

[9] Marques IL Monteiro LC de Souza L Bettiol H Sassaki CH de Assumpção Costa R . Gastroesophageal reflux in severe cases of Robin sequence treated with nasopharyngeal intubation. Cleft Palate Craniofac J. 2009;46(4):448-453. 19642776 10.1597/08-120.1

[10] Bull MJ Givan DC Sadove AM Bixler D Hearn D . Improved outcome in Pierre Robin sequence: effect of multidisciplinary evaluation and management. Pediatrics. 1990;86(2):294-301. 2371106

[11] Breik O Umapathysivam K Tivey D Anderson P . Feeding and reflux in children after mandibular distraction osteogenesis for micrognathia: a systematic review. Int J Pediatr Otorhinolaryngol. 2016;85:128-135. 27240511 10.1016/j.ijporl.2016.03.033

[12] Hyman PE . Gastroesophageal reflux: one reason why baby won't eat. J Pediatr. 1994;125(6 Pt 2):S103-S109. 10.1016/s0022-3476(05)82933-6 7983564

[13] Monasterio FO Molina F Berlanga F , et al. Swallowing disorders in Pierre Robin sequence: its correction by distraction. J Craniofac Surg. 2004;15(6):934-941. 15547378 10.1097/00001665-200411000-00009

[14] Kaditis AG Alonso Alvarez ML Boudewyns A , et al. ERS statement on obstructive sleep disordered breathing in 1- to 23-month-old children. Eur Respir J. 2017;50(6):1700985. doi:10.1183/13993003.00985-2017 29217599

[15] Kaditis AG Alonso Alvarez ML Boudewyns A , et al. Obstructive sleep disordered breathing in 2- to 18-year-old children: diagnosis and management. Eur Respir J. 2016;47(1):69-94. 26541535 10.1183/13993003.00385-2015

[16] Costa MA Tu MM Murage KP Tholpady SS Engle WA Flores RL . Robin sequence: mortality, causes of death, and clinical outcomes. Plast Reconstr Surg. 2014;134(4):738-745. 25357033 10.1097/PRS.0000000000000510

[17] Logjes RJH Haasnoot M Lemmers PMA , et al. Mortality in Robin sequence: identification of risk factors. Eur J Pediatr. 2018;177(5):781-789. 29492661 10.1007/s00431-018-3111-4 PMC5899115

[18] Spicuzza L Leonardi S La Rosa M . Pediatric sleep apnea: early onset of the ‘syndrome'? Sleep Med Rev. 2009;13(2):111-122. 19058983 10.1016/j.smrv.2008.07.001

[19] van Lieshout MJ Joosten KF Hoeve HL Mathijssen IM Koudstaal MJ Wolvius EB . Unravelling Robin sequence: considerations of diagnosis and treatment. Laryngoscope. 2014;124(5):E203-E209. 10.1002/lary.24437 24115087

[20] Paes EC van Nunen DP Speleman L , et al. A pragmatic approach to infants with Robin sequence: a retrospective cohort study and presence of a treatment algorithm. Clin Oral Investig. 2015;19(8):2101-2114. 10.1007/s00784-015-1407-6 PMC4592702 25680705

[21] Evans KN Sie KC Hopper RA Glass RP Hing AV Cunningham ML . Robin sequence: from diagnosis to development of an effective management plan. Pediatrics. 2011;127(5):936-948. 21464188 10.1542/peds.2010-2615 PMC3387866

[22] Resnick CM LeVine J Calabrese CE Padwa BL Hansen A Katwa U . Early management of infants with Robin sequence: an international survey and algorithm. J Oral Maxillofac Surg. 2019;77(1):136-156. 30599883 10.1016/j.joms.2018.05.031

[23] Logjes RJH MacLean JE de Cort NW , et al. Objective measurements for upper airway obstruction in infants with Robin sequence: what are we measuring? A systematic review. J Clin Sleep Med. 2021;17(8):1717-1729. 33960296 10.5664/jcsm.9394 PMC8656914

[24] Zaballa K Singh J Waters K . The management of upper airway obstruction in Pierre Robin sequence. Paediatr Respir Rev. 2023;45:11-14. 35987882 10.1016/j.prrv.2022.07.001

[25] Berry RB Budhiraja R Gottlieb DJ , et al. Rules for scoring respiratory events in sleep: update of the 2007 AASM manual for the scoring of sleep and associated events. Deliberations of the sleep apnea definitions task force of the American Academy of Sleep Medicine. J Clin Sleep Med. 2012;8(5):597-619. 23066376 10.5664/jcsm.2172 PMC3459210

[26] Caron C Pluijmers BI Joosten KFM , et al. Feeding difficulties in craniofacial microsomia: a multicenter retrospective analysis of 755 patients. J Craniomaxillofac Surg. 2018;46(10):1777-1782. 30158061 10.1016/j.jcms.2018.07.017

[27] Fredriks AM van Buuren S Burgmeijer RJ , et al. Continuing positive secular growth change in The Netherlands 1955-1997. Pediatr Res. 2000;47(3):316-323. 10709729 10.1203/00006450-200003000-00006

[28] Joosten KF Zwart H Hop WC Hulst JM . National malnutrition screening days in hospitalised children in The Netherlands. Arch Dis Child. 2010;95(2):141-145. 19414435 10.1136/adc.2008.157255

[29] Marcus CL Brooks LJ Draper KA , et al. Diagnosis and management of childhood obstructive sleep apnea syndrome. Pediatrics. 2012;130(3):e714-e755. 10.1542/peds.2012-1672 22926176

[30] Kaditis A Gozal D . Sleep studies for clinical indications during the first year of life: infants are not small children. Children (Basel). 2022;9(4). 10.3390/children9040523 PMC9025263 35455567

[31] Daftary AS Jalou HE Shively L Slaven JE Davis SD . Polysomnography reference values in healthy newborns. J Clin Sleep Med. 2019;15(3):437-443. 30853051 10.5664/jcsm.7670 PMC6411184

[32] Kanack MD Nakra N Ahmad I Vyas RM . Normal neonatal sleep defined: refining patient selection and interpreting sleep outcomes for mandibular distraction. Plast Reconstr Surg Glob Open. 2022;10(1):e4031. 10.1097/GOX.0000000000004031 PMC8769137 35070593

[33] Brockmann PE Poets A Poets CF . Reference values for respiratory events in overnight polygraphy from infants aged 1 and 3months. Sleep Med. 2013;14(12):1323-1327. 24211071 10.1016/j.sleep.2013.07.016

[34] Vézina K Mariasine J Young R , et al. Cardiorespiratory monitoring data during sleep in healthy Canadian infants. Ann Am Thorac Soc. 2020;17(10):1238-1246. 32678717 10.1513/AnnalsATS.201909-703OC

[35] Papastamelos C Panitch HB England SE Allen JL . Developmental changes in chest wall compliance in infancy and early childhood. J Appl Physiol (1985). 1995;78(1):179-184. 7713809 10.1152/jappl.1995.78.1.179

[36] Tan HL Kaditis AG . Phenotypic variance in pediatric obstructive sleep apnea. Pediatr Pulmonol. 2021;56(6):1754-1762. 33543838 10.1002/ppul.25309

[37] Kaditis A Kheirandish-Gozal L Gozal D . Pediatric OSAS: oximetry can provide answers when polysomnography is not available. Sleep Med Rev. 2016;27:96-105. 26146027 10.1016/j.smrv.2015.05.008

[38] Manica D Schweiger C Sekine L , et al. Association of polysomnographic parameters with clinical symptoms severity grading in Robin sequence patients: a cohort nested cross-sectional study. Sleep Med. 2018;43:96-99. 29482821 10.1016/j.sleep.2017.11.1136

[39] Buchenau W Wenzel S Bacher M Müller-Hagedorn S Arand J Poets CF . Functional treatment of airway obstruction and feeding problems in infants with Robin sequence. Arch Dis Child Fetal Neonatal Ed. 2017;102(2):F142-F146. 10.1136/archdischild-2016-311407 27435577

[40] Fahradyan A Azadgoli B Tsuha M Urata MM Francis SH . A single lab test to aid Pierre Robin sequence severity diagnosis. Cleft Palate Craniofac J. 2019;56(3):298-306. 29791187 10.1177/1055665618778400

[41] de Buys Roessingh AS Herzog G Hohlfeld J . Respiratory distress in Pierre Robin: successful use of pharyngeal tube. J Pediatr Surg. 2007;42(9):1495-1499. 17848237 10.1016/j.jpedsurg.2007.04.024

[42] Kwan JT Ebert BE Roby BB Scott AR . Detection of chronic hypoventilation among infants with Robin sequence using capillary blood gas sampling. Laryngoscope. 2021;131(12):2789-2794. 33914349 10.1002/lary.29594

[43] American Academy of Sleep Medicine. The International Classification of Sleep Disorders:(ICSD-3). American Academy of Sleep Medicine; 2014.

[44] Bütow KW Naidoo S Zwahlen RA Morkel JA . Pierre Robin sequence: subdivision, data, theories, and treatment - part 4: recommended management and treatment of Pierre Robin sequence and its application. Ann Maxillofac Surg. 2016;6(1):44-49. 27563606 10.4103/2231-0746.186136 PMC4979342

[45] Wagener S Rayatt SS Tatman AJ Gornall P Slator R . Management of infants with Pierre Robin sequence. Cleft Palate Craniofac J. 2003;40(2):180-185. 12605525 10.1597/1545-1569_2003_040_0180_moiwpr_2.0.co_2

[46] Marques IL de Sousa TV Carneiro AF Barbieri MA Bettiol H Pereira Gutierrez MR . Clinical experience with infants with Robin sequence: a prospective study. Cleft Palate Craniofac J. 2001;38(2):171-178. 11294545 10.1597/1545-1569_2001_038_0171_cewiwr_2.0.co_2

[47] Drago Marquezini Salmen IC Lazarini Marques I . In situ and home care nasopharyngeal intubation improves respiratory condition and prevents surgical procedures in early infancy of severe cases of Robin sequence. Biomed Res Int. 2015;2015:608905. 26273635 10.1155/2015/608905 PMC4529914

[48] Wilson AC Moore DJ Moore MH Martin AJ Staugas RE Kennedy JD . Late presentation of upper airway obstruction in Pierre Robin sequence. Arch Dis Child. 2000;83(5):435-438. 11040155 10.1136/adc.83.5.435 PMC1718546

[49] Smith MC Senders CW . Prognosis of airway obstruction and feeding difficulty in the Robin sequence. Int J Pediatr Otorhinolaryngol. 2006;70(2):319-324. 16112206 10.1016/j.ijporl.2005.07.003

[50] van den Elzen APM Semmekrot BA Bongers EMHF Huygen PLM Marres HAM . Diagnosis and treatment of the Pierre Robin sequence: results of a retrospective clinical study and review of the literature. Eur J Pediatr. 2001;160(1):47-53. doi:10.1007/s004310000646 11195018

[51] Maas C Poets CF . Initial treatment and early weight gain of children with Robin sequence in Germany: a prospective epidemiological study. Arch Dis Child Fetal Neonatal Ed. 2014;99(6):F491-F494. 10.1136/archdischild-2014-306472 25164557

[52] Lidsky ME Lander TA Sidman JD . Resolving feeding difficulties with early airway intervention in Pierre Robin sequence. Laryngoscope. 2008;118(1):120-123. 17975504 10.1097/MLG.0b013e31815667f3

[53] Daniel M Bailey S Walker K , et al. Airway, feeding and growth in infants with Robin sequence and sleep apnoea. Int J Pediatr Otorhinolaryngol. 2013;77(4):499-503. 23313433 10.1016/j.ijporl.2012.12.019

[54] Wan T Wang G Yang Y . The nutrition status of mild form Pierre Robin sequence before cleft palate repair: an analysis of 34 cases. Oral Surg Oral Med Oral Pathol Oral Radiol. 2014;118(1):43-46. 23312544 10.1016/j.oooo.2012.10.007

[55] Li HY Lo LJ Chen KS Wong KS Chang KP . Robin sequence: Review of treatment modalities for airway obstruction in 110 cases. Int J Pediatr Otorhinolaryngol. 2002;65(1):45-51. 12127222 10.1016/s0165-5876(02)00131-3

[56] Freezer NJ Bucens IK Robertson CF . Obstructive sleep apnoea presenting as failure to thrive in infancy. J Paediatr Child Health. 1995;31(3):172-175. 7669373 10.1111/j.1440-1754.1995.tb00779.x

[57] Paes EC Mink van der Molen AB Muradin MS , et al. A systematic review on the outcome of mandibular distraction osteogenesis in infants suffering Robin sequence. Clin Oral Investig. 2013;17(8):1807-1820. 10.1007/s00784-013-0998-z 23722462

[58] Cozzi F Totonelli G Frediani S Zani A Spagnol L Cozzi DA . The effect of glossopexy on weight velocity in infants with Pierre Robin syndrome. J Pediatr Surg. 2008;43(2):296-298. 18280277 10.1016/j.jpedsurg.2007.10.015

[59] Heaf DP Helms PJ Dinwiddie R Matthew DJ . Nasopharyngeal airways in Pierre Robin syndrome. J Pediatr. 1982;100(5):698-703. 7069530 10.1016/s0022-3476(82)80567-2

[60] Kolstad CK Senders CW Rubinstein BK Tollefson TT . Mandibular distraction osteogenesis: at what age to proceed. Int J Pediatr Otorhinolaryngol. 2011;75(11):1380–1384. 10.1016/j.ijporl.2011.07.032 21906821

[61] Sidman JD Sampson D Templeton B . Distraction osteogenesis of the mandible for airway obstruction in children. The Laryngoscope. 2001;111(7):1137-1146. doi:10.1097/00005537-200107000-00004 11568533

[62] Abramowicz S Bacic JD Mulliken JB Rogers GF . Validation of the GILLS score for tongue-lip adhesion in Robin sequence patients. J Craniofac Surg. 2012;23(2):382–386. doi:10.1097/SCS.0b013e318240fc7b 22421830

